# Communication Tapestry: Health Literacy Mediates Public Trust in Physician Health Information in Pakistani Public Hospitals

**DOI:** 10.3390/healthcare13030290

**Published:** 2025-01-31

**Authors:** Dake Wang, Talib Hussain, Wang Weiying

**Affiliations:** 1 School of Media and Communication, Shanghai Jiao Tong University, Shanghai 200240, China; dakewang@sjtu.edu.cn; 2 Department of Business Management, Karakoram International University, Diamer Campus, Gilgit 15100, Pakistan; talib.hussain@kiu.edu.pk; 3 Department of Media Management, University of Religions and Denominations, Qom 37491-13357, Iran; 4 China Research Institute for Science Popularization (CRISP), Beijing 100081, China

**Keywords:** healthcare trust, physician reputation, patient involvement, transparent communication, health literacy, prescribed medications, public hospitals, Pakistan

## Abstract

Background/Objectives: This study explores the multifaceted factors influencing public trust in healthcare services provided by doctors in public hospitals in Pakistan. The objective is to examine the relationships between various determinants such as doctors’ reputation and expertise, patient participation in decision-making, communication clarity, health literacy levels, and trust in prescribed medications to provide actionable insights for improving healthcare trust. Methods: A total of 550 patients from public hospitals were surveyed, and data were analyzed using Partial Least Squares Structural Equation Modeling (PLS-SEM). This approach enabled the identification of intricate relationships between the key factors influencing trust in healthcare services. Results: The findings indicate that patient participation in decision-making and transparent communication significantly enhance trust in prescribed medications. Additionally, health literacy emerged as a crucial factor, with higher levels of understanding leading to greater confidence in healthcare services. Conclusions: The study highlights the importance of patient-centered care, clear communication strategies, and health literacy initiatives in strengthening public trust in healthcare systems. Practical recommendations are provided for policymakers, healthcare professionals, and researchers to collaboratively improve healthcare service delivery and foster public confidence.

## 1. Introduction

Efficient physician–patient communication is the cornerstone of trust, essential for building confidence and ensuring adherence to medical recommendations [1]. The importance of this dynamic is heightened when considered in the context of Pakistan’s public hospitals, which continue to struggle with difficulties pertaining to the accessibility and quality of treatment [2]. Understanding the intricacies of communication between a physician and a patient becomes of the utmost importance in such an environment [3].

Aims and objectives of the research:

- Within the framework of Pakistani public hospitals, to systematically evaluate the influence that the reputation and experience of physicians have on the level of trust that patients have in the medications that they have been prescribed [4].
- To investigate the complex relationship that exists between patient participation in decision-making and patient trust in prescribed medications, with the goal of shedding light on the channels via which collaborative healthcare partnerships have an effect on trust dynamics [5].
- This study aims to analyze the role that open and honest communication plays in establishing patient confidence in prescribed medications, as well as to investigate the ways in which communication practices influence the establishment and maintenance of trust [6].
- In order to shed light on the processes via which health literacy influences trust dynamics, the purpose of this study is to investigate the function that health literacy plays as a mediator in the relationship between physician communication practices and patient trust in prescription pharmaceuticals [7].

This study significantly enhances the existing literature by delivering a thorough analysis of public trust in healthcare services in Pakistan, a largely unexamined context. By incorporating elements such as physician reputation, patient engagement, communication transparency, health literacy, and trust in prescribed medications, the research elucidates the intricate dynamics of trust. The innovative use of Partial Least Squares Structural Equation Modeling (PLS-SEM) facilitates the examination of complex interrelations among latent variables and the mediating effects of health literacy. This methodological framework introduces rigor and depth to the study, rectifying deficiencies in prior research that employed less advanced analytical methods [8,9].

## 2. Literature Review

Communication that is both effective and efficient between patients and their physicians is an essential component of the structure that is the provision of high-quality medical care. Its value extends far beyond the simple act of exchanging information; it is the foundation upon which trust, comprehension, and collaboration in the provision of care are constructed [10]. The paternalistic model, in which physicians assumed authoritative roles in decision-making, has given way to the contemporary paradigm of patient-centered care, which promotes shared decision-making and collaborative partnerships between patients and healthcare providers. This essential aspect of healthcare has undergone a transformation over the course of time, tracing a trajectory from the paternalistic model to the contemporary paradigm [7,11].

There is a continuous body of empirical research that demonstrates the enormous influence that communication has on the results of patient care across a wide range of healthcare organizations. It has been proven in a great number of research studies that there is a considerable association between effective communication and patient satisfaction, treatment adherence, and health outcomes [12,13].

This alliance is defined by trust, empathy, and mutual respect. Not only does this collaborative approach improve patient satisfaction, but it also fosters a sense of ownership and empowerment, which in turn enables patients to take an active role in the management of their own health [14,15].

### 2.1. Having Faith in Medical Care

When it comes to the complicated web that is the relationship between a patient and a physician, trust is an essential component that establishes the foundation for the relationship and extends its impact to encompass the wider landscape of healthcare delivery. It encompasses a fundamental confidence in the dependability, competency, and compassion of healthcare practitioners and organizations, which in turn shapes the actions, attitudes, and patterns of the healthcare utilization of patients [16,17].

Continuity of care emerges as a powerful promoter of trust, expressing the experiences of patients of constant and coordinated interactions with healthcare providers across the course of their treatment. Patients who have continuing relationships with their healthcare providers build a sense of familiarity, rapport, and mutual understanding with their providers. This helps to foster trust via the sharing of experiences and longitudinal treatment trajectories [18,19].

This research aims to analyze the overarching dynamics of patient–physician interactions, specifically the elements that affect trust in healthcare environments. The study encompasses elements of patient participation in medical decision-making, including dialogues regarding drug therapies; nevertheless, it primarily focuses on the relational and communicative factors that influence trust. It is recognized that specific decisions concerning medicine selection, dosage, and administration frequently entail minimal direct patient involvement. The research underscores the significance of shared decision-making in defining care objectives and cultivating trust, which transcends particular treatment options to include the entire quality of contact and communication between patients and physicians. [20]. These behaviors include adhering to treatment regimens, participating in preventive screenings, and adopting healthy lifestyle practices. A catalyst for patient empowerment, trust enables individuals to make educated decisions about their health and well-being with confidence and conviction. Trust serves as a catalyst for patient empowerment [21,22].

### 2.2. The Reputation and Experience of Physicians

The reputation and experience of physicians play a crucial role in establishing patient trust and satisfaction in healthcare environments. This encompasses not only the clinical skills and expertise of medical practitioners but also the perceptions and expectations shaped by patients’ interactions and experiences. [22,23].

While clinical competence, ensured through rigorous training and continuous professional development, is essential for physicians to deliver high-quality care, it does not directly translate into patient trust [24]. Research suggests that patients often assess trustworthiness based on observable and interpersonal factors, such as the physician’s ability to demonstrate empathy, clear communication, and attentiveness during consultations. Moreover, environmental elements, such as a welcoming and well-maintained clinic setting, also play a significant role in shaping patients’ perceptions of trust. These factors, though unrelated to clinical training, are critical for fostering a positive patient–doctor relationship [25,26].

### 2.3. The Role of Patient Involvement in the Decision-Making Process

The transition toward patient-centered care signifies a significant transformation in healthcare delivery, emphasizing the importance of fostering a trusting relationship that encourages patients to actively participate in decision-making processes. Beyond autonomy and empowerment, patient engagement also involves creating an environment where patients feel safe to share complete and accurate information with their healthcare providers. Trust plays a pivotal role in enabling patients to disclose sensitive or stigmatized details, such as health behaviors, the use of supplements, or embarrassing symptoms. This openness is critical for accurate diagnoses and appropriate treatment, highlighting the bidirectional nature of trust in enhancing both patient-centered care and clinical outcomes [27,28].

The existing body of empirical research repeatedly indicates that patient involvement has a significant influence on healthcare outcomes, patient satisfaction, and treatment adherence. Furthermore, patients who actively engage in the process of making decisions are more inclined to comply with treatment plans, execute suggested actions, and attain improved health results in the long run [29,30].

Moreover, inequalities in health literacy and the availability of information pose significant obstacles to successful patient engagement, especially in marginalized and vulnerable communities [31]. Individuals who possess a restricted level of health literacy may encounter difficulties in comprehending intricate medical information, effectively navigating many treatment alternatives, and advocating for their personal preferences. These challenges might further contribute to the existing gaps in healthcare access and results [32,33].

### 2.4. Clear and Open Communication

Transparent communication, which is grounded in ethical imperatives and patient-centered concepts, goes beyond the simple transmission of information [34]. Its purpose is to cultivate trust, improve patient comprehension, and facilitate collaborative decision-making in healthcare environments [35,36,37].

Nevertheless, the implementation of transparent communication is not devoid of obstacles and intricacies. Healthcare professionals may encounter feelings of uncertainty or worry when engaging in conversations about sensitive subjects or conveying bad information to patients. In a similar vein, physicians may encounter obstacles in their capacity to participate in open and unhurried discussion with patients due to perceived time restrictions and conflicting demands. This might restrict opportunities for transparent communication and shared decision-making [38,39].

In order to tackle these difficulties and encourage open and honest communication, healthcare institutions should allocate resources towards extensive training initiatives that specifically target communication proficiency, empathy, and the practice of sharing information. These programs provide physicians with the necessary tools and techniques to effectively negotiate intricate communication settings, cultivate empathy and comprehension, and establish trusted relationships with patients [40,41,42].

Inadequate health literacy represents a significant public health challenge, with far-reaching implications for individuals’ health outcomes, healthcare utilization patterns, and overall well-being. While studies consistently demonstrate that limited health literacy is associated with unfavorable health outcomes, higher healthcare costs, and reduced adherence to medical guidelines, it is crucial to recognize that these outcomes are influenced by a complex interplay of factors. This multifaceted approach underscores the importance of integrating health literacy as a vital component of holistic healthcare strategies [43,44,45].

In order to effectively tackle the obstacles presented by low health literacy, it is imperative for healthcare practitioners and organizations to adopt specific interventions that are designed to enhance health literacy and foster patient participation and empowerment. These interventions may involve creating materials in clear and understandable language, using visual aids, and conducting interactive educational sessions that are specifically designed to meet the requirements and preferences of patients. Healthcare practitioners can improve patients’ comprehension of health information and encourage them to actively participate in their healthcare decision-making by utilizing techniques such as effective communication, repetition, and reinforcement [46].

### 2.5. The Role of Health Literacy as a Mediator

The fundamental nature of health literacy’s mediating function highlights the significant significance of attending to patients’ informational requirements and enabling them to actively engage in their healthcare choices. Utilizing effective communication strategies, such as employing clear and concise explanations, incorporating visual aids, and implementing teach-back approaches, plays a crucial role in improving patient comprehension and involvement, ultimately leading to better health results [47].

Health literacy is pivotal in bridging the gap between individual patient interactions and broader healthcare system behaviors [48]. By prioritizing health literacy initiatives, healthcare professionals and organizations can address communication barriers, enhance collaborative decision-making, and foster greater patient trust and satisfaction. Additionally, cultivating a culture of transparency, openness, and patient-centered care empowers healthcare organizations to create environments that encourage patients to actively engage in their healthcare decisions and advocate for their needs effectively [49,50].

### 2.6. Integrating Social Science Perspectives on Trust in Healthcare Contexts

Trust has been extensively examined as an essential element in cultivating relationships, defined by attributes such as competence, integrity, and compassion. These qualities are especially pertinent in healthcare, where trust relies on patients’ judgments of physicians’ competence, ethical conduct, and sympathetic communication. Researchers have underscored that trust is contingent upon environment, influenced by human relationships, institutional structures, and cultural conventions. In healthcare, trust enhances effective communication and impacts adherence to medical recommendations and patient happiness. Our study contextualizes patients’ impressions within a broader theoretical framework by integrating various viewpoints, illustrating the connection between communication strategies and health literacy in shaping confidence in public healthcare organizations. This profound engagement with the literature fortifies the conceptual foundations of our study and amplifies its contribution to the area [51,52].

### 2.7. Identifying Research Gaps and Exploring Future Directions

An essential aspect that necessitates further examination is the efficacy of interventions aimed at enhancing the communication skills of physicians. Despite the acknowledgment of communication as a fundamental element of superior healthcare, there are insufficient data regarding the causal efficacy of particular communication training programs and tactics in improving physician–patient relations. Although connections exist between enhanced communication and improved patient outcomes, additional research is required to elucidate these associations and assess if specific communication interventions directly affect provider behaviors, patient trust, and the quality of treatment. Subsequent research must emphasize thorough evaluations of these treatments to ascertain their effects on patient and provider experiences, hence guiding the creation of customized training programs suitable for various clinical environments [53].

Addressing gaps in health literacy is a vital domain for study and intervention. Although health literacy is significantly correlated with patient outcomes, the causal mechanisms by which it affects healthcare experiences remain inadequately examined. Research should concentrate on assessing interventions designed to enhance health literacy, especially within disadvantaged and marginalized groups, to ascertain their direct impacts on patient empowerment, healthcare utilization, and overall health outcomes [54,55].

This study surpasses current approaches by using a multidimensional framework to investigate public trust in healthcare services, highlighting the interaction between physician communication techniques, health literacy, and patient participation. This research transcends prior models that often concentrate on single components by analyzing the intricate interplay of trust dynamics and their collective impact on confidence in healthcare providers and compliance with medical recommendations. Moreover, incorporating varied patient demographics and regional differences offers a more representative and inclusive viewpoint, rectifying shortcomings in previous research that were deficient in geographical and socioeconomic diversity. This work utilizes PLS-SEM to rigorously evaluate theoretical constructs and reveal hitherto underexamined mediation effects, including the function of health literacy as an intermediary between physician–patient communication and trust. This thorough methodology establishes the study as a notable contribution to the literature, providing practical insights for healthcare policymakers and practitioners to enhance trust-building efforts [56,57].

## 3. Methodology

The present study employed a cross-sectional research design to explore the dynamics of public trust in health information provided by physicians at public hospitals in Pakistan. A deliberate and structured sampling strategy was implemented to ensure the diversity and representativeness of the sample.

### 3.1. Sampling and Recruitment

The study recruited 550 participants from various regions of Pakistan. The key regions covered included Islamabad, Lahore, Karachi, Peshawar, Quetta, Skardu, and Gilgit. Participants were selected using probability sampling techniques to achieve demographic, geographic, and medical diversity [58].

The demographic factors considered included age, gender, socioeconomic status, educational attainment, and health conditions. Geographic diversity encompassed variations in urbanization levels, while medical categories were included to represent different healthcare utilization patterns. Inclusion criteria required participants to be aged 18 years or older, to have accessed public hospital services within the past six months, and to be capable of providing informed consent. Exclusion criteria included individuals under 18, those unable to provide informed consent, and those seeking care exclusively from private healthcare providers.

The sample size of 550 was calculated to ensure statistical power for robust data analysis. A total of 650 questionnaires were distributed, resulting in a response rate of 84.6%. Recruitment was conducted in diverse healthcare settings such as outpatient clinics, inpatient wards, and hospital waiting areas. Comparisons between respondents and non-respondents revealed no significant differences in demographic characteristics, ensuring the representativeness of the sample.

### 3.2. Instrument Design and Validation

Data were collected using a structured questionnaire designed to capture key constructs related to physician communication, health literacy, and public trust. The questionnaire was developed based on an extensive review of the literature and included validated scales addressing the following dimensions:

- Physician Reputation and Experience
- Patient Engagement in Decision-making
- Transparent Communication
- Health Literacy
- Trust in Healthcare Professionals

Cronbach’s alpha was used to assess internal consistency, and the questionnaire was refined based on pilot results to ensure its suitability for the target population.

### 3.3. Data Collection and Analysis

Data collection spanned 16 months, from 1 May 2023 to 31 August 2024. Participant responses were anonymized to maintain confidentiality and align with privacy standards. Raw data were organized and cleaned in Microsoft Excel 365, and then analyzed using SmartPLS 3 software for Structural Equation Modeling (SEM).

The SEM approach allowed the evaluation of complex relationships between variables and the testing of theoretical models. A Partial Least Squares (PLS) method was employed to estimate latent constructs, assess path coefficients, and evaluate model fit indices. Mediation analyses were conducted to explore the role of health literacy as a mediator between physician communication practices and patient outcomes. Bootstrapping techniques were applied to estimate indirect effects and validate mediating pathways.

### 3.4. Ethical Considerations

The study received ethical approval from the Ethics Committee of Karakoram International University (Ref. No. KIUDMR00116, dated 25 April 2023). Written informed consent was obtained from all participants in compliance with ethical and legal standards for human research. The confidentiality and anonymity of participant data were rigorously maintained throughout the research process [59,60].

### 3.5. Conceptual Model

- Conceptual Framework

The conceptual framework was developed based on hypotheses derived from the literature, outlining relationships between constructs such as communication, trust, and health literacy. The theoretical models informed the design and interpretation of SEM analyses, ensuring a structured approach to understanding the dynamics of public trust in healthcare.

- Theoretical framework and hypothesis development

The suitability of PLS-SEM was determined based on its causal predictive analysis capabilities and its flexibility to incorporate both reflecting and formative variables.

**Figure 1:**
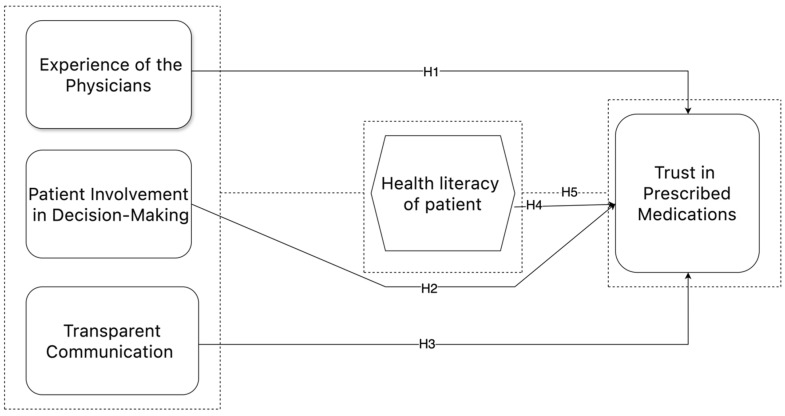
Conceptual model.

The objective of this work was to develop a conceptual model (as stated in Figure 1), that would shed light on the complex relationship between different latent variables and their related manifest variables. The aforementioned aspects were systematically classified into five unique categories, namely, construction-related, stakeholder-related, material-related, design-related, and external-related factors. Every factor was defined as an observable variable in the model, making it easier to evaluate through empirical analysis.

To examine the relationships proposed in the conceptual model, five hypotheses were generated in accordance with the research objectives.

Table 1 provides a thorough classification of the primary elements that impact public confidence in health information provided by physicians in public hospitals in Pakistan. It includes newly given codes for each sub-construct to aid in the methodical analysis of data. The factors under consideration encompass multiple dimensions that are essential for comprehending the dynamics between patients and healthcare providers. These dimensions include the Reputation and Experience of the Doctor (A), Patient Involvement in Decision-making (C), Transparent Communication (B), the Health Literacy of the Patient (E), and Trust in Prescribed Medications (D). Each factor contains distinct sub-constructs, including Doctor’s Reputation and Diagnostic Competence (A_1, A_2) under the Reputation and Experience of the Doctor, the Consideration of Patient Preferences and the Discussion of Financial Constraints (C_1, C_3) under Patient Involvement in Decision-making, Transparency about Financial Relationships and the Discussion of Conflicts of Interest (B_1, B_3) under Transparent Communication, Confidence in Understanding Medical Information and Seeking Additional Information (E_1, E_3) under the Health Literacy of Patient, and Trust in Prescribed Medications and Confidence in Medication Effectiveness (D_1, D_2). The aforementioned codes offer a systematic framework for examining the complex interconnections among many components and sub-constructs, hence illuminating the elements that influence public confidence in health information within the healthcare setting of Pakistan.

## 4. Data Analysis

The study employed Smart-PLS, Version 4.0, the latest version, a commonly used software for doing Structural Equation Modeling (SEM) in exploratory research [61]. Structural Equation Modeling (SEM) enables the investigation of intricate associations between identified and underlying factors, rendering it highly suitable for the analysis of multiple constructs within the healthcare domain [62]. We utilized Structural Equation Modeling (SEM) to evaluate the influence of observed factors on underlying constructs, including the Doctor’s Reputation and Experience, Patient Engagement in Decision-making, Clear Communication, Patients’ Health Literacy, and Trust in Prescribed Medications. Structural Equation Modeling (SEM) facilitates the concurrent examination of numerous associations within a singular model, hence facilitating a thorough comprehension of the determinants impacting public confidence in health information provided by physicians in public hospitals in Pakistan [63].

### 4.1. Data Screening and Cleaning

The study employed stringent and methodical data screening and cleansing protocols to uphold the quality and integrity of the gathered data.

Initially, all submissions underwent meticulous examination to detect absent, incomplete, or contradictory entries. This method maintained the dataset’s representativeness while reducing potential biases.

The outliers were evaluated contextually to ascertain their legitimacy as data points or errors, therefore, ensuring that valid data were not unduly discarded.

This involved juxtaposing participant replies with established logical criteria and verifying that values remained within expected ranges. Demographic data, including age and income, were examined for validity.

Moreover, duplicate items were detected and eliminated by cross-referencing participant identifiers and timestamps to eradicate redundancy in the dataset. Open-ended replies, when relevant, were categorized and standardized to enhance analysis.

The meticulous procedure of data screening and cleansing guaranteed the dataset’s integrity and dependability, establishing a robust basis for ensuing statistical analysis and interpretation.

### 4.2. Measurement Model

The Measurement Model in Partial Least Squares Structural Equation Modeling (PLS-SEM) is a fundamental element used to analyze the connections between latent constructs and their observable indicators. It enables the evaluation of the dependability, agreement, and differentiation of the measurement tools used in the study. In this step, the focus is on closely examining the connections between hidden concepts and the measurements used to represent them, evaluating the consistency of the measurement scales, and verifying the accuracy of the concepts being studied. The Measurement Model establishes a thorough grasp of the connections between variables and their consequences for the study hypotheses through careful analysis and improvement.

### 4.3. Evaluation of Outer Measurement Model

The section pertaining to the evaluation of the outer Measurement Model offers a thorough examination of the dependability, internal coherence, convergent validity, and discriminant validity of the observable variables and latent constructs in the research. The reliability and internal consistency of the constructs were confirmed using standardized outer loadings, Cronbach’s alpha, Composite Reliability (CR), and Average Variance Extracted (AVE). Furthermore, the research conducted in this study aimed to show convergent validity by proving that each latent construct accounted for more than 50% of the variance observed in the variables. Additionally, the study sought to establish discriminant validity by comparing the correlations between constructs to their respective Average Variance Extracted (AVE) values [64].

Table 2 displays the reliability and validity measures for the main variables in the study on public confidence in health information from physicians in Pakistani public hospitals. The evaluation of each primary variable is based on Cronbach’s alpha, Composite Reliability (rho_a), Composite Reliability (rho_c), and Average Variance Extracted (AVE).

The Health Literacy of Patients demonstrates strong reliability and validity, as evidenced by a Cronbach’s alpha coefficient of 0.904, Composite Reliability (rho_a) of 0.905, a Composite Reliability (rho_c) of 0.929, and an Average Variance Extracted (AVE) of 0.723. This demonstrates the robust internal consistency and dependability of the Measurement Model for this variable.

The Patient Involvement in Decision-making measure demonstrates high levels of reliability and validity, as evidenced by a Cronbach’s alpha coefficient of 0.911, a Composite Reliability (rho_a) of 0.912, a Composite Reliability (rho_c) of 0.934, and an Average Variance Extracted (AVE) of 0.738. These values demonstrate strong internal consistency and reliability, as well as confirm the convergent validity of this variable.

The Doctor’s Reputation and Experience exhibit strong reliability and validity, as evidenced by a Cronbach’s alpha coefficient of 0.888, a Composite Reliability (rho_a) of 0.888, a Composite Reliability (rho_c) of 0.922, and an Average Variance Extracted (AVE) of 0.748. This indicates a high level of internal consistency and dependability, as well as excellent convergent validity for this variable.

Transparent Communication demonstrates strong reliability and validity, as evidenced by a Cronbach’s alpha coefficient of 0.908, a Composite Reliability (rho_a) of 0.909, a Composite Reliability (rho_c) of 0.931, and an Average Variance Extracted (AVE) of 0.731. These values demonstrate robust internal consistency and reliability, as well as convergent validity for this variable.

The level of Trust in Prescribed Medications, although still displaying adequate levels of reliability and validity, has significantly lower values in comparison to the other variables. The Cronbach’s alpha coefficient is 0.864, the Composite Reliability (rho_a) is 0.861, the Composite Reliability (rho_c) is 0.904, and the Average Variance Extracted (AVE) is 0.655. Although the results are slightly lower, the variable still demonstrates sufficient internal consistency and reliability, as well as convergent validity.

### 4.4. Discriminant Validity

The Heterotrait-Monotrait (HTMT) ratio analysis offers useful insights into the discriminant validity of the constructs within the Measurement Model. The analysis is essential for verifying the differentiation between the constructs in the study, which demonstrates the efficacy of the Measurement Model in accurately representing the underlying concepts.

An analysis, as shown in Table 3, of the HTMT ratios across all components uncovers significant trends. The construct of Health Literacy demonstrates satisfactory discriminant validity with all other components, as seen by HTMT ratios ranging from 0.544 to 0.723. This indicates that Health Literacy is separate from Patient Involvement in Decision-making, the Reputation and Experience of the Doctor, Transparent Communication, and Trust in Prescribed Medications.

Similarly, the Patient Involvement in Decision-making concept shows adequate discriminant validity when compared to all other constructs. The HTMT ratios for this comparison range from 0.238 to 0.593. This suggests that Patient Involvement in Decision-making can be differentiated from factors such as Health Literacy, the Doctor’s Reputation and Experience, Transparent Communication, and Trust in Prescribed Medications.

In addition, the Reputation and Experience of the Doctor demonstrates satisfactory discriminant validity with all other constructs, as indicated by HTMT ratios ranging from 0.238 to 0.614. This indicates that the reputation and experience of the doctor are separate from Health Literacy, Patient Involvement in Decision-making, Transparent Communication, and Trust in Prescribed Medications.

In addition, Transparent Communication has satisfactory discriminant validity with all other constructs, as indicated by HTMT ratios ranging from 0.255 to 0.615. Transparent Communication can be differentiated from Health Literacy, Patient Involvement in Decision-making, the Reputation and Experience of the Doctor, and Trust in Prescribed Medications.

Finally, the Trust in Prescribed Medications construct demonstrates satisfactory discriminant validity with all other constructs, as evidenced by HTMT ratios ranging from 0.593 to 0.723. This indicates that Trust in Prescribed Medications is separate from Health Literacy, Patient Involvement in Decision-making, Reputation and Experience of the Doctor, and Transparent Communication.

The Fornell–Larcker criterion in Table 4 is a crucial tool for evaluating the discriminant validity between the constructs in the Measurement Model. Discriminant validity guarantees that each construct in the model is separate from the others, showing that they measure distinct features of the phenomenon being studied. In this analysis, each cell in the table indicates the correlation between two constructs, while the diagonal elements display the square root of the Average Variance Extracted (AVE) for each construct.

Based on the Health Literacy of Patient construct, the diagonal value of 0.850 indicates that 85% of the variation in the observed variables can be traced to the underlying construct. This shows a good level of reliability. Furthermore, the off-diagonal values provide insight into the relationships between Health Literacy and other categories. For example, the correlation coefficient between Health Literacy and Patient Involvement in Decision-making is 0.504, which is less than the square root of the Average Variance Extracted (AVE) for Health Literacy. This finding supports the discriminant validity of Health Literacy.

Regarding the Patient Involvement in Decision-making construct, the diagonal value of 0.859 signifies that 85.9% of the variation in the observed variables can be accounted for by the underlying construct. The off-diagonal values depict the associations between Patient Involvement in Decision-making and other factors. As an illustration, the correlation between the Reputation and Experience of the Doctor is 0.214, which is less than the square root of the Average Variance Extracted (AVE) for Patient Involvement in Decision-making. This suggests that there is discriminant validity.

Similarly, the diagonal value of 0.865 for the Reputation and Experience of the Doctor construct indicates a high level of dependability. This means that 86.5% of the variance in the observed variables can be assigned to this construct. The off-diagonal values indicate the correlations with other variables, such as Transparent Communication (0.230) and Trust in Prescribed Medications (0.539), which are lower than the square root of the AVE for Reputation and Experience of the Doctor, thus proving its discriminant validity.

The diagonal value of 0.855 for the Transparent Communication construct shows that 85.5% of the variance in the observed variables can be explained by the underlying construct. The non-diagonal elements indicate correlations with other variables, such as Trust in Prescribed Medications (0.548), which is less than the square root of the Average Variance Extracted (AVE) for Transparent Communication, confirming its ability to distinguish from other constructs.

The Trust in Prescribed Medications construct has a diagonal value of 0.809, indicating that 80.9% of the variation in the observed variables can be accounted for by the construct. The off-diagonal values exhibit correlations with other dimensions, such as the Health Literacy of Patient (0.648), that are lower than the square root of the Average Variance Extracted (AVE) for Trust in Prescribed Medications, demonstrating discriminant validity.

The Fornell–Larcker criterion table validates the discriminant validity of each concept in the Measurement Model, demonstrating that they accurately assess different parts of the phenomenon being studied. This improves the dependability and authenticity of the research results, instilling trust in the precision of the measuring model.

Table 5 presents the cross-loading values for each sub-construct inside the main constructs of the study model. The cross-loading values indicate the correlations between the observed variables and the latent constructs, enabling an evaluation of the construct validity. The bolded values in the table highlight the strongest correlations between each observed variable and its corresponding latent construct, confirming construct validity and the alignment of sub-constructs with their respective main variables.

The cross-loading values of the Reputation and Experience of the Doctor construct reveal the specific contribution of each sub-construct to the overall construct. As an illustration, the Doctor’s Reputation construct exhibits cross-loading values that range from 0.416 to 0.441. These values indicate a moderate to strong link with the Reputation and Experience of the Doctor construct. Similarly, other components such as Doctor’s Diagnostic Competence, the Length of Practice, Second Opinion Seeking, and Recommendations from Friends/Family show significant correlations with the Reputation and Experience of the Doctor component, with cross-loading values ranging from 0.404 to 0.429.

Regarding the Transparent Communication construct, the cross-loading values provide insight into the connections between each sub-construct and the overall construct. The variables of Transparency about Financial Relationships, the Influence of Transparency on Trust, the Discussion of Conflicts of Interest, the Value of Transparency in Communication, and Trust Based on Disclosure of Financial Ties show moderate to strong correlations with the Transparent Communication construct. The cross-loading values range from 0.446 to 0.862.

In the Patient Involvement in Decision-making construct, cross-loading values offer insights into the connections between each sub-construct and the overall construct. The Patient Involvement in Decision-making construct is significantly correlated with factors such as Consideration of Patient Preferences, the Level of Involvement in Decision-making, the Discussion of Financial Constraints, Comfort in Expressing Preferences, and Seeking Second Opinions. The cross-loading values for these factors range from 0.157 to 0.881.

In relation to the construct of Trust in Prescribed Medications, cross-loading values show the degree to which each sub-construct contributes to the overall construct. The constructs of Trust in Prescribed Medications, Confidence in Medication Effectiveness, Confidence in Medication Safety, Trust Influenced by Doctor’s Decision-making, and Trust and Adherence to Treatment Plan have moderate to strong correlations with the Trust in Prescribed Medications construct. The cross-loading values range from 0.387 to 0.874.

Finally, in the context of the Health Literacy of Patient construct, cross-loading values provide clarity on the connections between each sub-construct and the main construct. The variables Confidence in Understanding Medical Information, Difficulty in Understanding Medical Terminology, Seeking Additional Information, Comfort in Discussing Health Concerns, and the Interpretation of Medical Test Results show strong correlations with the Health Literacy of Patient construct. The cross-loading values range from 0.381 to 0.883.

**Figure 2:**
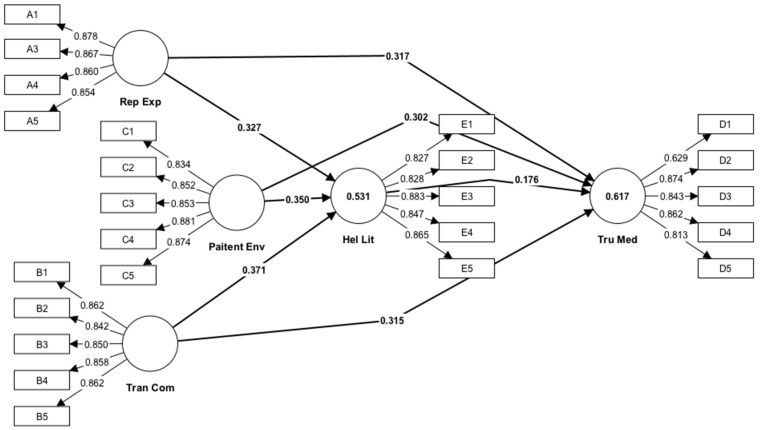
Path coefficient.

Table 6 and Figure 2 display the path coefficients, which indicate the magnitude and direction of the connections between the latent constructs in the Structural Equation Model (SEM). 

The path coefficient of 0.176 indicates a positive correlation between the Health Literacy of Patients and their Faith in Prescription Medications. These findings suggest a positive correlation between Health Literacy and Trust in Prescription Drugs. The *t* statistics value of 3.951 is statistically significant (*p* < 0.05), suggesting that the occurrence of this association by chance is highly unusual.

Regarding the route coefficients related to Patient Engagement in Decision-making, the coefficients of 0.350 and 0.302 for its links with Patients’ Health Literacy and Trust in Prescribed Medications, respectively, demonstrate significant positive correlations. The findings indicate that more Patient Participation in Decision-making is linked to higher levels of Health Literacy and Trust in Prescription Drugs. Both path coefficients exhibit high T statistics values, indicating a high level of statistical significance.

Furthermore, the path coefficients for the Doctor’s Reputation and Experience exhibit substantial positive correlations with both the Health Literacy of Patients (0.327) and their Faith in Recommended Pharmaceuticals (0.317). The results suggest that patients exhibit elevated levels of Health Literacy and Confidence in Drugs when their physicians possess a robust reputation and considerable expertise. 

Transparent Communication is positively associated with Health Literacy of Patients and Faith in Given Medications, as indicated by path coefficients of 0.371 and 0.315, respectively. These findings indicate that implementing Transparent Communication techniques can result in increased levels of Health Literacy and Trust in Prescription Medications among patients.

The level of Trust in Prescribed Medications, although still displaying adequate levels of reliability and validity, has significantly lower values in comparison to the other variables. The Cronbach’s alpha coefficient is 0.864, the Composite Reliability (rho_a) is 0.861, the Composite Reliability (rho_c) is 0.904, and the Average Variance Extracted (AVE) is 0.655. Although the results are slightly lower, the variable still demonstrates sufficient internal consistency and reliability, as well as convergent validity.

The confidence intervals presented in the table provide valuable information about the accuracy and dependability of the calculated path coefficients in the Structural Equation Model (SEM). 

The association between the Health Literacy of Patients and their Trust in Prescribed Medications is indicated by the confidence interval for the path coefficient (0.087, 0.260). This means that we may be 95% confident that the actual value of the path coefficient lies within this range. The interval serves as a way to quantify the uncertainty surrounding the calculated coefficient of 0.176. It indicates a moderate to strong positive correlation between Health Literacy and Trust in Prescription Medications.

The confidence intervals for the path coefficients related to Patient Involvement in Decision-making, Doctor’s Reputation and Experience, and Transparent Communication provide information about the feasible range of values for these coefficients. The intervals provided, such as 0.290 to 0.407 for patient engagement in decision-making and its link to the Health Literacy of Patients, serve to illustrate the level of the precision of the estimates and provide a measure of confidence in the relationships being investigated.

In general, confidence intervals as shown in Table 7 serve to provide a range of values within which the true coefficients are likely to be found, thus helping to put the predicted path coefficients into context. They function as a beneficial instrument for evaluating the strength of the model’s estimates and comprehending the uncertainty linked to the connections between the constructs being studied.

Table 8 displays the cumulative indirect impacts of three primary characteristics—Patient Engagement in Decision-making, Doctor’s Reputation and Experience, and Transparent Communication—on Trust in Prescribed Medications.

The original sample indicates that there is an estimated effect size of 0.062 when considering the impact of Patient Involvement in Decision-making on Trust in Prescribed Drugs. This indicates that for each incremental rise of one unit in Patient Participation in Decision-making, there is a corresponding increase of 0.062 in the level of trust placed in recommended medications. The standard deviation (STDEV) of 0.017 represents the degree of variation in the impact seen throughout the sample. The *t* statistics value of 3.722 indicates that the effect is statistically significant (*p* < 0.001), implying that it is highly improbable to have happened randomly.

In the original sample, the estimated indirect effect of the Doctor’s Reputation and Experience on Faith in Recommended Pharmaceuticals is 0.057. This suggests that there is a positive correlation between the Reputation and Experience of the Doctor and the level of Trust in Prescription Medications. Specifically, for every one-unit rise in the Reputation and Experience of the Doctor, there is a corresponding increase of 0.057 in the level of Trust in Prescribed Medications. The *t* statistics value of 3.599 suggests that this impact is statistically significant at a very low level of probability (*p* < 0.001).

The original sample assessed the indirect effect of clear communication on Trust in Prescribed Drugs to be 0.065. This indicates that for each incremental rise of one unit in Transparent Communication, there is a corresponding gain of 0.065 units in Trust towards Recommended Medications. Like the other effects, the T statistics value of 3.841 demonstrates that this effect is statistically significant (*p* < 0.001).

In summary, these findings indicate that Patient Participation in Decision-making, the Doctor’s Reputation and Experience, and Clear Communication have substantial indirect impacts on Trust in Prescribed Medications. This underscores the significance of these factors in shaping patient Trust and Confidence in their Prescribed Treatments.

**Figure 3:**
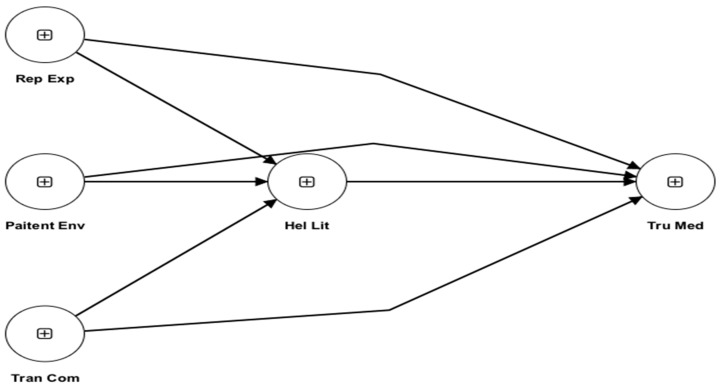
Q^2^predict.

Q^2^predict as shown in Table 9 and Figure 3, is a crucial metric used in Partial Least Squares Structural Equation Modeling (PLS-SEM) to assess the predictive significance of the model for each construct. In this study, the Q^2^predict values offer insights into the degree to which the model accurately predicts the observed values of the endogenous variables, namely, the Health Literacy of Patients and Trust in Prescribed Pharmaceuticals.

The sub-constructs of confidence in comprehending medical information, difficulty in understanding medical terminology, seeking additional information, comfort in discussing health concerns, and the interpretation of medical test results have Q^2^predict values ranging from 0.339 to 0.394, which contribute to the Health Literacy of Patients. These values demonstrate that the PLS-SEM model effectively predicts patients’ health literacy levels by explaining a substantial amount of the variance in these dimensions. The model’s high Q^2^predict values indicate that it has strong predictive relevance for various dimensions of health literacy.

The Q^2^predict values for Trust in Prescribed Medications vary from 0.353 to 0.409 across different sub-constructs. These sub-constructs include Trust in Prescribed Medications, Confidence in Medication Effectiveness, Confidence in Medication Safety, Trust Influenced by Doctor’s Decision-making, and Trust and Adherence to Treatment Plan. These values demonstrate that the model effectively captures a significant amount of the variation in Trust in Prescription Medications, showing a high level of predictive significance. The elevated Q^2^predict values indicate that the model accurately forecasts patients’ Trust Levels in Prescribed Medications by considering many aspects associated with medication efficacy, safety, physician decision-making, and adherence to treatment programs.

The Q^2^predict values indicate that the PLS-SEM model has a strong ability to predict both the Health Literacy of Patients and their Faith in Prescribed Medications. The findings demonstrate the model’s capacity to predict patients’ Health Literacy levels and Faith in Prescribed Medications with accuracy, using specific sub-constructs. This provides useful insights for understanding and improving patient outcomes in healthcare settings.

### 4.5. Findings and Discussions

This facilitates the maintenance of a constant tone throughout the content. This section tries to provide a comprehensive summary of the study’s objectives, with a specific focus on the importance of examining the factors that impact public trust in health information in Pakistani public hospitals. Specifically, this section will focus on the importance of the performed investigation. Furthermore, we explore the prospects of trust in healthcare-related environments. Furthermore, we offer a description of the study methodology, which relies on the application of Partial Least Squares Structural Equation Modeling (PLS-SEM) to examine the relationships among the main variables. This is achieved by establishing the basis for this in the introductory section of our findings, which also helps to prime the reader for a more comprehensive examination of the study’s results.

The presentation of the results includes a detailed discussion of the empirical findings obtained from the investigations conducted on our data. The data we provide for each notion includes both descriptive information and inferential analysis. Furthermore, the researchers examine the external loadings of each construct, as well as the discriminant validity and the Heterotrait-Monotrait ratio (HTMT). This enables a thorough understanding of the accuracy and consistency of the Measurement Model. Furthermore, we provide an extensive array of tables and figures that effectively illustrate the data, facilitating comprehension. These are the goods that our clients have access to. This comprehensive presentation of the data provides readers with an understanding of the empirical relationships between the variables, as well as the statistical significance of the findings.

In order to evaluate the results, it is essential to conduct an inquiry into the relevance of the findings and the consequences of the empirical research that was conducted. In this work, we examine the significance of key path coefficients by specifically examining the magnitude and direction of connections between independent variables, variables that mediate those connections, and variables that are influenced by those interactions. By placing the statistical results inside the theoretical framework and research aims, we can get insight into the factors that impact public trust in healthcare services. This enables us to illuminate the mechanisms that influence public trust. As a result, we acquired a more profound understanding of the factors that influence the public’s trust in healthcare services. These approaches incorporate patient participation, transparent and truthful communication, and enhanced health literacy as key components. Furthermore, we examine the intricate method via which these factors interact, considering the various varied moderating effects and channels of influence that exist. Our objective is to discover the key factors that contribute to the development of trust in Pakistani public hospitals through the use of rigorous analytical methods. The implementation of our study will serve as the means by which this target will be achieved.

Our findings are situated within the broader research conducted on trust in hospital settings throughout the years. This comparison is conducted within the context of comparing it to earlier studies. Within this part, we will commence by undertaking an inquiry into the relevant empirical studies and theoretical frameworks. Subsequently, we shall juxtapose our findings with the already established discoveries. The objective of this undertaking is to identify the regions where the two sets of data align, share similarities, and differ from each other. In this study, we provide insights on how applicable and reliable our findings are in different healthcare settings and cultural contexts. The attainment of this objective is accomplished by synthesizing information gathered from a diverse array of data sources. Furthermore, we provide a thorough examination of the many methodological approaches and theoretical assumptions that have been employed previously, emphasizing both the positive impacts and the drawbacks of the research conducted in the process. These actions are performed in addition to this one. By conducting this comparative study, we have not only enhanced our understanding of the mechanisms that establish trust in the healthcare industry, but we have also identified potential areas for future research investigation using this method.

The explanation of the most significant discoveries leads to a thorough investigation of the corresponding outcomes and their immediate ramifications. In this study, we will focus on the practical implications that main path coefficients have for the practice, policy, and research in the healthcare industry. The substantial importance of significant path coefficients is examined, with a particular focus on the practical implications of these coefficients. Through analyzing the interrelationships among Patient Engagement, Transparent Communication, Health Literacy, and Trust in Healthcare Services, we can uncover the mechanisms that account for the impact of these factors on patient-centered care and the provision of high-quality healthcare. This enables us to elucidate the mechanisms that are accountable for the contributions made by these elements. This allows us to acquire a more profound understanding of the mechanisms responsible for these contributions. In addition, we aim to gain a deeper understanding of the intricate dynamics involved in the interactions between patients and physicians by examining the factors that influence the establishment of trust in this study. With this comprehensive explanation, we aim to clarify the fundamental elements of our research. Furthermore, we will provide stakeholders in the healthcare industry concrete solutions that may be implemented.

The section on implications provides a concise overview of the most important findings and a thorough analysis of the broader consequences that these discoveries have on the healthcare industry’s practice, policy, and research. This part also covers an analysis of the ramifications that arise from those discoveries. The aim of this section is to examine the real-world consequences of our findings and propose strategies that can be applied in public hospitals in Pakistan to improve patient-centered care, encourage transparent communication, and enhance health literacy. We also engage in a discussion regarding the consequences for healthcare policy, and we strongly advocate for incorporating patient-centered approaches and communication training into the ongoing changes in the healthcare system. Furthermore, a conversation is conducted to address the consequences for healthcare policy. In addition, we undertake an examination of the broader societal consequences of trust in healthcare, considering its influence on patient outcomes, the disparities that exist in healthcare, and the effects on public health. Through our active engagement in this comprehensive dialogue, we are optimistic about our ability to contribute to the development of evidence-based strategies and policies that prioritize patient empowerment and foster trust in healthcare settings.

Prior to offering an impartial and objective assessment of the degree to which the findings can be applied to a broader context, it is crucial to acknowledge the limitations of the study. This will enable a more precise and unbiased assessment. This article discusses the limitations of the study, such as its cross-sectional design and the dependence on self-reported metrics. The article discusses several methodological limits, and these examples illustrate some of them. We consider the constraints connected with the environment as an additional issue to consider. The limitations of this study include the specific attributes of the sample being studied, as well as the broader sociocultural context of public hospitals in Pakistan. Furthermore, we assess the study’s findings by carefully considering the potential influence of biases and confounding factors that may have affected the determination of the results. To elucidate the limitations of our research and potential avenues for further exploration, we can openly and truthfully discuss these boundaries. This is accomplished with the objective of providing valuable information.

Here are some ongoing observations and proposals for ongoing research:

Based on the information we obtained from our analysis, we offer suggestions for future research directions and improvements in methodology. The report contains these recommendations. These guidelines have been formulated based on the findings of our investigation. We support conducting longitudinal research to examine the temporal dynamics of trust formation in healthcare environments, as well as the enduring impacts of patient-centered interventions. We wholeheartedly embrace this belief. We are highly passionate about having a solid conviction in this matter. In addition, we recommend doing a qualitative study to examine the perspectives and real-life experiences of patients on their trust in the services provided by healthcare professionals. This investigation must be undertaken to satisfy our recommendation. In addition, we advocate for doing comparative research concurrently in multiple cultural contexts and healthcare settings to enhance the generalizability of the findings. This would be carried out to improve the overall applicability of the results. Furthermore, we stress the importance of carrying out intervention studies to evaluate the effectiveness of trust-building strategies and communication therapies in genuine healthcare environments that exist in the actual world. The objective of these notions is to promote future research activities that will enhance our comprehension of trust in healthcare and facilitate the development of evidence-based methods.

### 4.6. Study Limitations

This study is limited by its cross-sectional design, which restricts the ability to infer causality between variables. Additionally, the reliance on self-reported data may introduce response bias, and the findings may not fully capture the perspectives of patients in private healthcare settings or those unable to access healthcare services. Future studies could address these limitations by employing longitudinal designs and expanding the sample to include diverse healthcare contexts.

### 4.7. Implications of Study

The implications of this study are substantial for both healthcare practice and policy. The research identifies critical aspects, including Patient Participation, Transparent Communication, and Health Literacy, offering actionable insights to enhance trust in healthcare services inside public hospitals. The results indicate that cultivating trust by patient-centered care and transparent communication can improve patient outcomes and compliance with medical recommendations. Healthcare officials and experts are urged to incorporate these tactics into hospital practices, especially in Pakistan, to enhance public trust in healthcare services.

## 5. Conclusions

This study enhances the existing research on trust in healthcare by pinpointing critical factors that affect public trust in public hospitals in Pakistan. Utilizing Partial Least Squares Structural Equation Modeling (PLS-SEM), our work offers significant insights into the intricate mechanisms influencing trust in healthcare environments. When healthcare providers take into account patient choices and engage in transparent communication regarding treatment options, individuals exhibit increased trust in their recommended medications. Moreover, the reputation and experience of healthcare experts, especially their diagnostic acumen and years of practice, were identified as essential elements in fostering confidence. The study underscores the significance of health literacy in enabling patients to comprehend medical information and interpret test findings, hence enhancing their confidence in healthcare services.

These findings hold considerable implications for healthcare practice, policy, and research in Pakistan and analogous situations. The research underscores the necessity for patient-centered methodologies that promote collaborative decision-making, clear communication, and health education. Healthcare professionals and politicians can leverage these findings to formulate tailored initiatives that empower patients, improve their comprehension of health-related information, and foster trust in healthcare systems. Furthermore, our study advocates for sustained initiatives to tackle healthcare inequities, enhance cultural competence, and fortify patient–provider relationships to guarantee fair access to high-quality healthcare for all demographics.

This study provides significant insights; nonetheless, numerous limitations must be recognized. The cross-sectional methodology restricts the capacity to infer causal associations, necessitating future research to employ longitudinal or experimental methodologies to corroborate the identified causal connections. The generalizability of these findings may be limited by the particular attributes of the study population and the sociocultural environment of public hospitals in Pakistan. Further study is required to enhance the generalizability of the findings by examining the dynamics of trust across other healthcare environments and cultural contexts.

This study elucidates the determinants of trust in healthcare services and presents pragmatic solutions for improving patient-centered care and fostering trust in public hospitals in Pakistan. By emphasizing patient engagement, clear communication, and health literacy, healthcare providers and policymakers may cultivate trust, elevate patient satisfaction, and ultimately improve health outcomes. Ongoing study and collaboration are crucial for enhancing our comprehension of trust in healthcare and developing more resilient, patient-centered healthcare systems.

### Future Proposed Study

Future research could explore the impact of these factors in similar socioeconomic contexts across different regions to further validate and enhance the generalizability of the findings. By examining how trust in healthcare is shaped in diverse settings, researchers can gain a deeper understanding of the universality and contextual variability of the identified factors, ultimately leading to more effective, adaptable strategies for improving patient trust and communication in healthcare systems worldwide.

## Figures and Tables

**Table 1:** Construct code development.

Code	Factors
Reputation and Experience of the Doctor	
A_1	To what extent does the reputation of your doctor influence your trust in the prescribed medication?
A_2	How confident are you in the doctor’s ability to accurately diagnose and prescribe effective medications?
A_3	Does the length of time your doctor has been practicing influence your level of trust in their prescribed medications?
A_4	How likely are you to seek a second opinion when prescribed medication by a less-experienced doctor?
A_5	How much importance do you place on the doctor’s professional reputation when considering prescribed medications?
Patient Involvement in Decision-making	
C_1	To what extent did your doctor consider your preferences and concerns when prescribing the medication?
C_2	How satisfied are you with the level of involvement your doctor allows you in the decision-making process for your treatment plan?
C_3	Were the potential financial constraints related to the prescribed medication discussed with you?
C_4	How comfortable do you feel expressing your preferences and concerns about prescribed medications to your doctor?
C_5	How likely are you to seek a second opinion if your doctor insists on a medication that you are uncomfortable taking?
Transparent Communication	
B_1	To what extent do you believe your doctor is transparent about any potential financial relationships with pharmaceutical companies?
B_2	How important is the transparency of your doctor’s financial relationships in influencing your trust in prescribed medications?
B_3	Did your doctor openly discuss any potential conflicts of interest before prescribing the medication?
B_4	How much do you value transparency in the doctor’s communication about the pharmaceutical industry’s influence on prescription practices?
B_5	How likely are you to trust prescribed medications if your doctor discloses any financial relationships with pharmaceutical companies?
Health Literacy of Patient	
E_1	How confident are you in understanding medical information provided by healthcare professionals?
E_2	How often do you encounter difficulty in understanding medical terminology or instructions provided by doctors or pharmacists?
E_3	How frequently do you seek additional information or clarification about your health condition or prescribed medication from reliable sources (e.g., healthcare professionals, reputable websites)?
E_4	To what extent do you feel comfortable discussing your health concerns or asking questions during medical consultations?
E_5	How confident are you in interpreting and understanding medical test results or reports provided by healthcare professionals?
Trust in Prescribed Medications	
D_1	To what extent do you trust the medications prescribed by your doctor?
D_2	How confident are you in the effectiveness of the medications prescribed by your doctor in treating your medical condition?
D_3	How confident are you in the safety of the medications prescribed by your doctor, in terms of potential side effects and risks?
D_4	To what extent does your trust in the medications prescribed by your doctor depend on your confidence in the doctor’s decision-making process?
D_5	How does your level of trust in prescribed medications influence your adherence to the prescribed treatment plan?

**Table 2:** Construct reliability and validity.

Main Variables	Cronbach’s Alpha	Composite Reliability (rho_a)	Composite Reliability (rho_c)	Average Variance Extracted (AVE)
Health Literacy of Patient	0.904	0.905	0.929	0.723
Patient Involvement in Decision-making	0.911	0.912	0.934	0.738
Reputation and Experience of the Doctor	0.888	0.888	0.922	0.748
Transparent Communication	0.908	0.909	0.931	0.731
Trust in Prescribed Medications	0.864	0.861	0.904	0.655

**Table 3:** Heterotrait-Monotrait ratio (HTMT)—matrix.

	Health Literacy of Patient	Patient Involvement in Decision-Making	Reputation and Experience of the Doctor	Transparent Communication	Trust in Prescribed Medications
Health Literacy of Patient					
Patient Involvement in Decision-making	0.555				
Reputation and Experience of the Doctor	0.544	0.238			
Transparent Communication	0.579	0.248	0.255		
Trust in Prescribed Medications	0.723	0.593	0.614	0.615	

**Table 4:** Fornell–Larcker criterion.

	Health Literacy of Patient	Patient Involvement in Decision-Making	Reputation and Experience of the Doctor	Transparent Communication	Trust in Prescribed Medications
Health Literacy of Patient	0.850				
Patient Involvement in Decision-making	0.504	0.859			
Reputation and Experience of the Doctor	0.487	0.214	0.865		
Transparent Communication	0.526	0.226	0.230	0.855	
Trust in Prescribed Medications	0.648	0.529	0.539	0.548	0.809

**Table 5:** Cross loadings.

Main Variables	Sub Constructs	Health Literacy of Patient	Patient Involvement in Decision-Making	Patient Involvement in Decision-Making	Transparent Communication	Trust in Prescribed Medications
Reputation and Experience of the Doctor	Doctor’s Reputation	0.416	0.193	0.878	0.187	0.469
	Doctor’s Diagnostic Competence	0.429	0.449	0.874	0.399	0.438
	Length of Practice	0.404	0.193	0.867	0.197	0.466
	Second Opinion Seeking	0.424	0.213	0.86	0.203	0.478
	Recommendations from Friends/Family	0.441	0.142	0.854	0.208	0.453
Transparent Communication	Transparency about Financial Relationships	0.472	0.217	0.19	0.862	0.466
	Influence of Transparency on Trust	0.478	0.175	0.218	0.842	0.453
	Discussion of Conflicts of Interest	0.46	0.211	0.202	0.85	0.506
	Value of Transparency in Communication	0.406	0.186	0.202	0.858	0.467
	Trust Based on Disclosure of Financial Ties	0.425	0.176	0.169	0.862	0.446
Patient Involvement in Decision-making	Consideration of Patient Preferences	0.402	0.834	0.157	0.197	0.466
	Level of Involvement in Decision-making	0.418	0.852	0.19	0.182	0.429
	Discussion of Financial Constraints	0.465	0.853	0.176	0.191	0.45
	Comfort in Expressing Preferences	0.429	0.881	0.188	0.191	0.437
	Second Opinion Seeking Comfortability	0.449	0.874	0.207	0.21	0.488
Trust in Prescribed Medications	Trust in Prescribed Medications	0.457	0.434	0.399	0.438	0.629
	Confidence in Medication Effectiveness	0.469	0.444	0.445	0.453	0.874
	Confidence in Medication Safety	0.436	0.422	0.425	0.463	0.843
	Trust Influenced by Doctor’s Decision-making	0.455	0.387	0.449	0.435	0.862
	Trust and Adherence to Treatment Plan	0.455	0.432	0.445	0.406	0.813
Health Literacy of Patient	Confidence in Understanding Medical Information	0.827	0.406	0.381	0.433	0.535
	Difficulty in Understanding Medical Terminology	0.828	0.453	0.416	0.447	0.587
	Seeking Additional Information	0.883	0.428	0.428	0.459	0.555
	Comfort in Discussing Health Concerns	0.847	0.444	0.423	0.429	0.522
	Interpretation of Medical Test Results	0.865	0.411	0.421	0.464	0.551

**Table 6:** Path coefficients.

	Original Sample (O)	Sample Mean (M)	Standard Deviation (STDEV)	*t* Statistics (|O/STDEV|)	*p* Values
Health Literacy of Patient → Trust in Prescribed Medications	0.176	0.174	0.044	3.951	0.000
Patient Involvement in Decision-making → Health Literacy of Patient	0.350	0.350	0.029	11.957	0.000
Patient Involvement in Decision-making → Trust in Prescribed Medications	0.302	0.302	0.030	9.986	0.000
Reputation and Experience of the Doctor → Health Literacy of Patient	0.327	0.327	0.031	10.571	0.000
Reputation and Experience of the Doctor → Trust in Prescribed Medications	0.317	0.317	0.032	9.894	0.000
Transparent Communication → Health Literacy of Patient	0.371	0.372	0.029	12.925	0.000
Transparent Communication → Trust in Prescribed Medications	0.315	0.316	0.033	9.623	0.000

**Table 7:** Confidence intervals.

	Original Sample (O)	Sample Mean (M)	2.5%	97.5%
Health Literacy of Patient → Trust in Prescribed Medications	0.176	0.174	0.087	0.260
Patient Involvement in Decision-making → Health Literacy of Patient	0.350	0.350	0.290	0.407
Patient Involvement in Decision-making → Trust in Prescribed Medications	0.302	0.302	0.243	0.363
Reputation and Experience of the Doctor → Health Literacy of Patient	0.327	0.327	0.266	0.387
Reputation and Experience of the Doctor → Trust in Prescribed Medications	0.317	0.317	0.253	0.379
Transparent Communication → Health Literacy of Patient	0.371	0.372	0.316	0.427
Transparent Communication → Trust in Prescribed Medications	0.315	0.316	0.252	0.381

**Table 8:** Total indirect effects.

	Original Sample (O)	Sample Mean (M)	Standard Deviation (STDEV)	T Statistics (|O/STDEV|)	*p* Values
Patient Involvement in Decision-making → Trust in Prescribed Medications	0.062	0.061	0.017	3.722	0.000
Reputation and Experience of the Doctor → Trust in Prescribed Medications	0.057	0.057	0.016	3.599	0.000
Transparent Communication → Trust in Prescribed Medications	0.065	0.065	0.017	3.841	0.000

**Table 9:** Q^2^predict.

Main Variable	Sub Constructs	Q^2^predict	PLS-SEM_RMSE	PLS-SEM_MAE	LM_RMSE	LM_MAE
Health Literacy of Patient (Moderating Variable)	Confidence in Understanding Medical Information	0.339	0.766	0.616	0.767	0.612
	Difficulty in Understanding Medical Terminology	0.394	0.777	0.617	0.767	0.608
	Seeking Additional Information	0.393	0.78	0.636	0.774	0.629
	Comfort in Discussing Health Concerns	0.382	0.799	0.649	0.787	0.638
	Interpretation of Medical Test Results	0.384	0.795	0.639	0.784	0.629
Trust in Prescribed Medications (Dependent Variable)	Trust in Prescribed Medications	0.353	0.788	0.629	0.778	0.618
	Confidence in Medication Effectiveness	0.409	0.764	0.61	0.776	0.621
	Confidence in Medication Safety	0.39	0.785	0.633	0.782	0.627
	Trust Influenced by Doctor’s Decision-making	0.364	0.794	0.634	0.788	0.629
	Trust and Adherence to Treatment Plan	0.375	0.777	0.636	0.773	0.631

## Data Availability

The data will be available on request.
